# Regional variation of underlying kidney diseases in children undergoing chronic kidney replacement therapy around the globe

**DOI:** 10.1007/s00467-025-07096-3

**Published:** 2025-12-08

**Authors:** Dagmara Borzych-Dużałka, Marjolein Bonthuis, Uma Ali, Yok-Chin Yap, Michael Manno, Yihui Zhai, Reyner Loza, Seema Hashmi, Naye Choi, Kenza Soulami, Judith Exantus, Mohamed S. Al Riyami, Sameh Mabrouk, Marbella Ma Angeles, Cristina Zelaya-Camacho, Henny Adriani Puspitasari, Syed Saimul Huque, Francisco Cano, Kar Hui Ng, Sevcan A. Bakkaloğlu, Jerome Harambat, Kitty J. Jager, Bradley A. Warady, Franz Schaefer

**Affiliations:** 1 Department of Pediatrics, Nephrology and Hypertension, Medical University of Gdańsk, Gdańsk, Poland; 2 Department of Medical Informatics, Amsterdam UMC, ESPN/ERA Registry, University of Amsterdam, Amsterdam, The Netherlands; 3 Amsterdam Public Health, Quality of Care, Amsterdam, The Netherlands; 4 Lilavati Hospital and Research Centre, Mumbai, India; 5 Hospital Tunku Azizah, Kuala Lumpur, Malaysia; 6 Canadian Institute for Health Information (CIHI), Toronto, Canada; 7 Children’s Hospital of Fudan University, Shanghai, China; 8 Cayetano Heredia Hospital, Lima, Peru; 9 Sindh Institute of Urology and Transplantation (SIUT), Karachi, Pakistan; 10 Seoul National University College of Medicine, Seoul National University, Children’s Hospital, Seoul, Republic of Korea; 11 Pediatric Nephrology, Casablanca, Morocco; 12 Department of Pediatrics, Faculty of Medicine and Pharmacy, State University of Haiti, State University Hospital of Haiti, Port-Au-Prince, Haiti; 13 Pediatric Nephrology, The Royal Hospital, Muscat, Oman; 14 Faculty of Medicine Ibn El Jazzar, Department of Pediatrics Hospital Sahloul, University of Sousse, Sousse, Tunisia; 15 National Kidney and Transplant Institute, Quezon City, The Philippines; 16 FUNDANIER - Foundation for Children With Kidney Diseases, Guatemala City, Guatemala; 17 Department of Child Health, Faculty of Medicine Universitas Indonesia, Dr. Cipto Mangunkusumo General Hospital, Jakarta, Indonesia; 18 Department of Pediatric Nephrology, Bangladesh Medical University, Dhaka, Bangladesh; 19 Department of Paediatric Nephrology, Samina Masud Santa, Bangladesh Medical University, Dhaka, Bangladesh; 20 Luis Calvo Mackenna Children’s Hospital, University of Chile, Santiago, Chile; 21 Department of Pediatrics, Yong Loo Lin School of Medicine National University of Singapore, Singapore, Singapore; 22 Faculty of Medicine, Department of Pediatrics, Division of Pediatric Nephrology, Gazi University, Ankara, Turkey; 23 Pediatric Nephrology Unit, Bordeaux University Hospital, Bordeaux, France; 24 Children’s Mercy Kansas City, Kansas City, MO USA; 25 Center for Pediatrics and Adolescent Medicine, University Hospital Heidelberg, Heidelberg, Germany

**Keywords:** Primary kidney disease, Kidney failure, Children, Regional variation

## Abstract

**Background:**

There is a scarcity of information regarding the distribution of the diseases leading to kidney failure (KF) in children living in the emerging world. We used registry data to provide a global overview of the underlying disease spectrum in children commencing kidney replacement therapy (KRT).

**Methods:**

We analyzed KF causes among 23,620 children and adolescents commencing maintenance KRT in 80 countries, using data from the IPNA Global KRT Registry (including ESPN/ERA Registry), the International Pediatric Dialysis Network (IPDN), the United States Renal Data System (USRDS), and the Australia and New Zealand Dialysis and Transplant Registry (ANZDATA). The analysis considered geographic region, country-level gross national income (GNI), average annual temperature, and patient age.

**Results:**

Marked regional differences were observed in the distribution of KF causes. Immune-mediated glomerulopathies (GP) were most common in Southeast Asia, hereditary nephropathies in the Middle East, Africa, and Europe, and systemic GP in Northeast Asia and Latin America. In 14% of cases the cause of KF was unknown, with the highest proportion in Northeast Asia. Disease patterns were also influenced by countries’ GNI and average yearly temperature; immune-mediated GP accounted for 43% of diagnoses in low-income countries and were more frequent in warmer climates. Among younger children, congenital anomalies of the kidney and urinary tract (CAKUT) and hereditary nephropathies were the predominant cause of KF, whereas adolescents more commonly presented with immune-mediated GP.

**Conclusion:**

There is significant global variability in the spectrum of diseases leading to pediatric KF, partially attributable to genetic, environmental, and macroeconomic factors.

**Graphical Abstract:**

**Figure:**
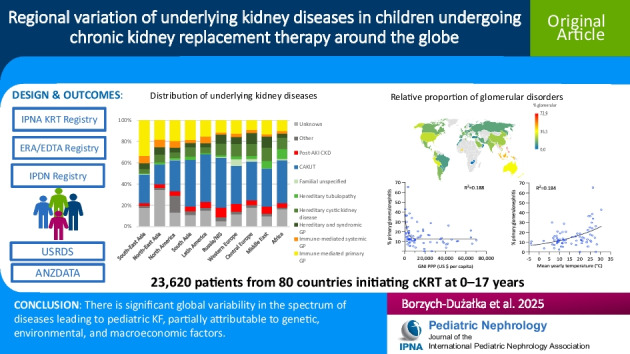
A higher resolution version of the Graphical abstract is available as Supplementary information

**Supplementary Information:**

The online version contains supplementary material available at 10.1007/s00467-025-07096-3.

## Introduction

The etiologies of pediatric kidney failure (KF) have been extensively studied in high-income regions, particularly in North America, Europe, Japan, and Australia/New Zealand. In these regions, congenital anomalies of the kidney and urinary tract (CAKUT) are the leading cause of KF, accounting for 30–60% of cases, followed by glomerular diseases (mainly focal segmental glomerulosclerosis (FSGS)), hereditary diseases including ciliopathies, and systemic conditions like lupus nephritis [1, 2].

Much less is known about the etiologies of KF in children living in low- and middle-income countries (LMIC). Literature is largely limited to single-center or regional reports, which may not be generalizable [3–5]. Some evidence suggests that children in emerging countries may face a higher prevalence of infectious and environmental etiologies, including various forms of post-infectious glomerulonephritis, systemic vasculitis and hemolytic uremic syndrome. Also, congenital obstructive uropathies may go undiagnosed or untreated due to limited access to prenatal imaging and perinatal care [6, 7].

The lack of systematic data collection is a major barrier to epidemiological research in low- and middle-income countries. According to a worldwide survey performed by the International Pediatric Nephrology Association (IPNA) in 2017, national registries for both dialysis and kidney transplantation in children were established in only 51 countries, covering just one third of the global child population [8]. The lack of systematic monitoring of a large part of the global pediatric population hampers quantitative assessment of the causes of KF, treatment access, management practices and clinical outcomes and slows the development of lifesaving, cost-efficient kidney replacement therapy (KRT) programs in many parts of the globe. To address this critical knowledge gap, the International Pediatric Nephrology Association Kidney Replacement Therapy (IPNA KRT) Registry was established in 2018 as a global registry collecting prospective population-based data on the demographics and etiologies of KF, KRT modality choices and patient and technique outcomes in children [9].

The aim of the current analysis, combining data from the IPNA KRT Registry, the ESPN/ERA Registry, the United States Renal Data System (USRDS), and the Australia and New Zealand Dialysis and Transplant Registry (ANZDATA), was to examine and compare the underlying causes of KF in children across diverse global regions. This comparative approach is hoped to enable a better understanding of regional differences in maintenance KRT, highlight disparities in diagnostic patterns and healthcare infrastructure, and inform global strategies for prevention, early detection and management of pediatric KF.

## Methods

### Data sources

Data were extracted from the International Pediatric Nephrology Association Kidney Replacement Therapy (IPNA KRT) Registry including ESPN/ERA Registry data (80 countries), the International Pediatric Dialysis Network (IPDN) (7 countries), published information from the United States Renal Data System (USRDS), and the Australian and New Zealand Dialysis and Transplant (ANZDATA) annual reports [9–13].

The global IPNA KRT registry collects population-based national data on children receiving either hemodialysis, peritoneal dialysis, or a kidney transplant. Data excerpts are provided by existing national and regional registries. In countries without established registries, pediatric KRT centers use an online data entry platform (www.ipna-registry.org) [9]. For the analysis presented here, 38 countries without established national registries used the online data entry system, 29 countries provided data via the ESPN/ERA Registry and 3 extracted data from national registry databases. Primary kidney diseases (PKD) were classified using the ERA-EDTA PKD coding system (Supplemental Table 1) [14].

**Table 1:** Countries by geographic region and number of patients 0–17 at KRT start per country (N = 80) and region (N = 10)

	Africa (n = 7)	Latin America (n = 12)	Western Europe (n = 14)	Eastern and Central Europe (n = 17)	Russia and Newly Independent States (n = 5)	Middle East (n = 7)	Northeast Asia (n = 3)	South Asia (n = 4)	South-East Asia & Oceania (n = 8)	North America And Caribbean (n = 3)
No. of patients by region	N = 240	N = 1,201	N = 6,033	N = 3,455	N = 1,929	N = 1,170	N = 1,086	N = 1710	N = 1,571	N = 5,227
No. of patients per country	Burkina Faso (7) Egypt (1) Morocco (140) Nigeria (4) Tunisia (81) Uganda (1) Zambia (6)	Argentina (209) Bolivia (16) Brazil (171) Chile (235) Colombia (43) Guatemala (79) Haiti (11) Mexico (27) Nicaragua (25) Peru (342) Paraguay (18) Uruguay (25)	Austria (269) Denmark (207) Finland (215) France (2210) Germany (415) Greece (63) Israel (125) Italy (222) Malta (6) Norway (186) Portugal (285) Spain (1218) Switzerland (231) UK (382)	Albania (33) Bosnia & Herzegovina (58) Croatia (112) Bulgaria (77) Cyprus (17) Czech Rep (104) Estonia (10) Hungary (49) Latvia (14) Lithuania (53) Macedonia (18) Poland (1111) Romania (317) Serbia (126) Slovenia (42) Slovakia (107) Turkey (1207)	Armenia (14) Belarus (139) Georgia (24) Russia (1411) Ukraine (341)	Jordan (123) Syria (214) Iran (622) Lebanon (2) Oman (14) Saudi Arabia (66) United Arab Emirates (129)	China (849) Hong Kong (70) South Korea (167)	Bangladesh (107) India (1230) Sri Lanka (55) Pakistan (317)	Indonesia (57) Laos (11) Malaysia (989) Philippines (133) Singapore (61) Vietnam (8) Australia/New Zealand (312)**	USA (4162)* Canada (1038) Puerto Rico (27)

^*^USRDS data, **ANZDATA

The International Pediatric Dialysis Network (IPDN) Registry prospectively collects clinical data from children commencing maintenance hemodialysis or peritoneal dialysis [11]. The IPDN disease classification system is presented in Supplemental Table 1.

For the IPNA and IPDN registries, the registry protocols were approved by the relevant institutional review boards at each participating center and/or country, in accordance with local requirements. The ESPN/ERA Registry provided data based on a trilateral agreement involving the IPNA registry, the ESPN/ERA Registry, and participating country. Written informed consent from parents or guardians, and patient assent when applicable, were obtained.

For pediatric patients with KF receiving KRT in the United States data were extracted from the USRDS report [12]. In the US, pediatric nephrologists are required to complete a Centers for Medicare & Medicaid (CMS) Medical Evidence Report containing initial demographic data and follow-up data. The United Network for Organ Sharing (UNOS) collects data on kidney transplantation and is reported in the USRDS database as well. The USRDS kidney diagnoses classification is depicted in Supplemental Table 1.

ANZDATA is a voluntary database to which patients who reside in Australia or New Zealand and receive chronic KRT are reported through a Web portal [13]. The ANZDATA kidney disease classification system is shown in Supplemental Table 1.

### Categorization of disease etiologies

For this study, PKD were classified into six main disease categories of interest and 10 subcategories: 1**) Congenital Anomalies of the Kidney and Urinary Tract** including kidney dysplasia, obstructive uropathy, reflux nephropathy and other CAKUT; **2) Immune-mediated glomerulopathies** including primary and secondary glomerulopathies; **3) Hereditary/familial nephropathies** including ciliopathies, familiar and syndromic glomerulopathies, hereditary tubulopathies and not further specified familial diseases; **4) post-AKI CKD** including thrombotic microangiopathies; **5) other** including tubulointerstitial nephritis, hematologic-oncologic and post-traumatic disease; **6) unknown**. For a complete list of diseases under each category, please see Supplemental Table 1.

### Regional and GNI and annual temperature categorization

Geographic regions were defined in accordance with the regional boards of the International Society of Nephrology: Africa, Central Europe, Latin America, Middle East, Russia and Newly Independent States (NIS), North America and the Caribbean, South Asia, Northeast Asia, Southeast Asia, and Western Europe (Table 1). Countries were classified as low, low-middle, upper-middle, and high-income countries based on World Word Bank data (https://data.worldbank.org) using quartiles of Gross National Income (GNI) in the calendar year of KRT initiation [15].

To assess the impact of climatic factors on the relative prevalence of disorders leading to KF, average yearly temperature was calculated by averaging the minimum and maximum daily temperatures in the country, averaged for the years 1991–2020, from the World Bank Group, derived from raw gridded climatologies of the Climatic Research Unit [16].

### Analysis dataset

All reported patients with KF who commenced chronic KRT at 0 to 17 years of age were included in the present analysis.

Patient-level data on date of birth, start date of KRT and PKD were extracted from the IPNA and IPDN registries, while age category and PKD were available from the USRDS and ANZDATA reports. The IPNA KRT Registry included incident patients who initiated KRT between 1997 and 2025, whereas the IPDN dataset comprised patients who began KRT between 2007 and 2025. The USRDS report included incident KRT patients from 2018 to 2022, while the ANZDATA report covered the period from 2017 to 2022. For a complete list of date of enrolment by region and country, please see Supplemental Table 2.

**Table 2:** Subjects receiving KRT by diagnostic category and geographic region

	Total	Africa	Latin America	Western Europe	Central/Eastern Europe	Russia/NIS	Middle East	Northeast Asia	South Asia	Southeast Asia, Australia, Oceania	North America	P
Total, N	*23,620*	*240*	*1,201*	*6,033*	*3,455*	*1,929*	*1,170*	*1,086*	*1,709*	*1,570*	*5,227*	
CAKUT	8,250 (35)	97 (41)	541 (45)	2,167 (36)	1,249 (36)	899 (47)	418 (36)	202 (19)	749 (44)	401 (25)	1,527 (30)	< 0.0001
Kidney dysplasia	4,089 (50)	35 (36)	301 (55)	1,283 (59)	418 (33)	420 (47)	162 (39)	160 (79)	320 (43)	205(51)	782 (51)	< 0.0001
Obstructive uropathy	2,241 (27)	20 (21)	128 (24)	518 (24)	399 (32)	284 (31)	66 (16)	13 (6)	209 (28)	95 (24)	509 (33)	< 0.0001
Reflux nephropathy	1,208 (15)	29 (30)	60 (11)	280 (13)	262 (21)	143 (16)	82 (20)	28 (14)	117 (16)	61 (15)	146 (9)	< 0.0001
Other CAKUT	715 (8)	13 (13)	52 (10)	86 (4)	170 (14)	52 (6)	108 (26)	1 (1)	103 (14)	40 (10)	90 (6)	< 0.0001
Familial/hereditary NP	4,785 (20)	60 (25)	124 (10)	1,653 (27)	941 (27)	426 (22)	353 (30)	175 (16)	276 (16)	159 (10)	618 (12)	< 0.0001
Cystic kidney disease	2,107 (44)	25 (42)	63 (51)	691 (42)	392 (42)	215 (50)	147 (42)	79 (45)	149 (54)	58 (36)	288 (47)	< 0.0001
Hereditary and syndromic GP	1,565 (32)	9 (15)	47 (38)	411 (25)	362 (38)	161 (38)	122 (35)	76 (43)	69 (25)	81 (51)	227 (37)	< 0.0001
Tubulopathies	577 (12)	23 (38)	14 (11)	169 (10)	95 (10)	32 (8)	79 (22)	18 (11)	55 (20)	12 (7)	80 (13)	< 0.001
Familial unspecified	536 (11)	3 (5)	0	382 (23)	92 (10)	18 (4)	5 (1)	2 (1)	3 (1)	8 (5)	23 (4)	< 0.001
Immune-mediated GP Primary Systemic	4,705 (20) 3,644 (77) 974 (13)	30 (13) 24 (80) 6 (20)	257 (21) 184 (72) 73 (28)	927 (15) 743 (80) 184 (20)	402 (12) 312 (78) 90 (22)	257 (13) 218 (85) 39 (15)	178 (15) 153 (86) 25 (14)	274 (25) 197 (61) 77 (28)	361 (21) 313 (87) 48 (13)	684 (43) 496 (83) 101 (17)	1,335 (26) 1004 (75) 331 (25)	< 0.0001 < 0.0001 < 0.0001
Post-AKI (TTP, toxic, Infectious) TTP	1,283 (5) 833 (64)	10 (4) 3 (33)	68 (6) 58 (85)	425 (7) 270 (63)	159 (5) 108 (67)	176 (9) 150 (85)	76 (6) 53 (69)	38 (4) 24 (63)	75 (4) 44 (59)	38 (2) 20 (52)	219 (4) 103 (47)	< 0.0001 < 0.0001
Other (tubulointerstitial, trauma, oncologic, surgical loss)	1,302 (6)	4 (2)	29 (2)	156 (2)	79 (2)	70 (4)	30 (2)	20 (2)	57 (3)	28 (2)	829 (17)	< 0.0001
Unknown/uncertain	3,294 (14)	39 (16)	182 (15)	705 (12)	625 (18)	101 (5)	115 (10)	377 (35)	191 (11)	260 (17)	699 (12)	< 0.0001

### Statistical analysis

Descriptive statistics were used to summarize results as median (interquartile range) for continuous variables and number (%) for categorical variables and analyzed using SAS 9.4. Chi-square tests were used to compare categorical variables such as etiology frequencies across different regions, countries and income groups. Non-linear regression analysis was utilized to evaluate relationships between continuous variables like gross national income per capita, average yearly temperature, and the proportion of specific KF etiologies.

Data were analyzed on regional and country-level. For the latter analyses, countries with at least 6 patients in a particular category were included. Statistical significance was set at *p* < 0.05.

## Results

### Regional distribution of kidney failure etiologies

KF etiology was extracted from data of 23,620 children and adolescents aged 0–17 at KRT initiation, across 80 countries and 10 world regions (Table 2).

**Fig. 1:**
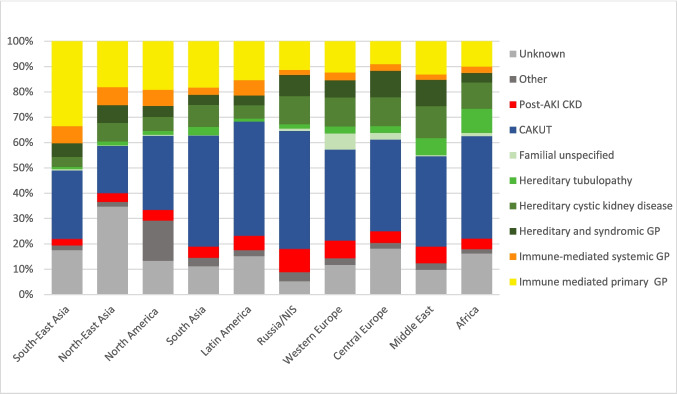
Distribution of primary kidney diseases causing kidney failure by global regions

Globally, the predominant underlying etiologies of KF were CAKUT (35%), hereditary nephropathies (20%), and immune-mediated glomerulopathies (20%) (Table 2, Fig. 1). In 14% of cases, the cause of KF was reported as unknown.

**Fig. 2:**
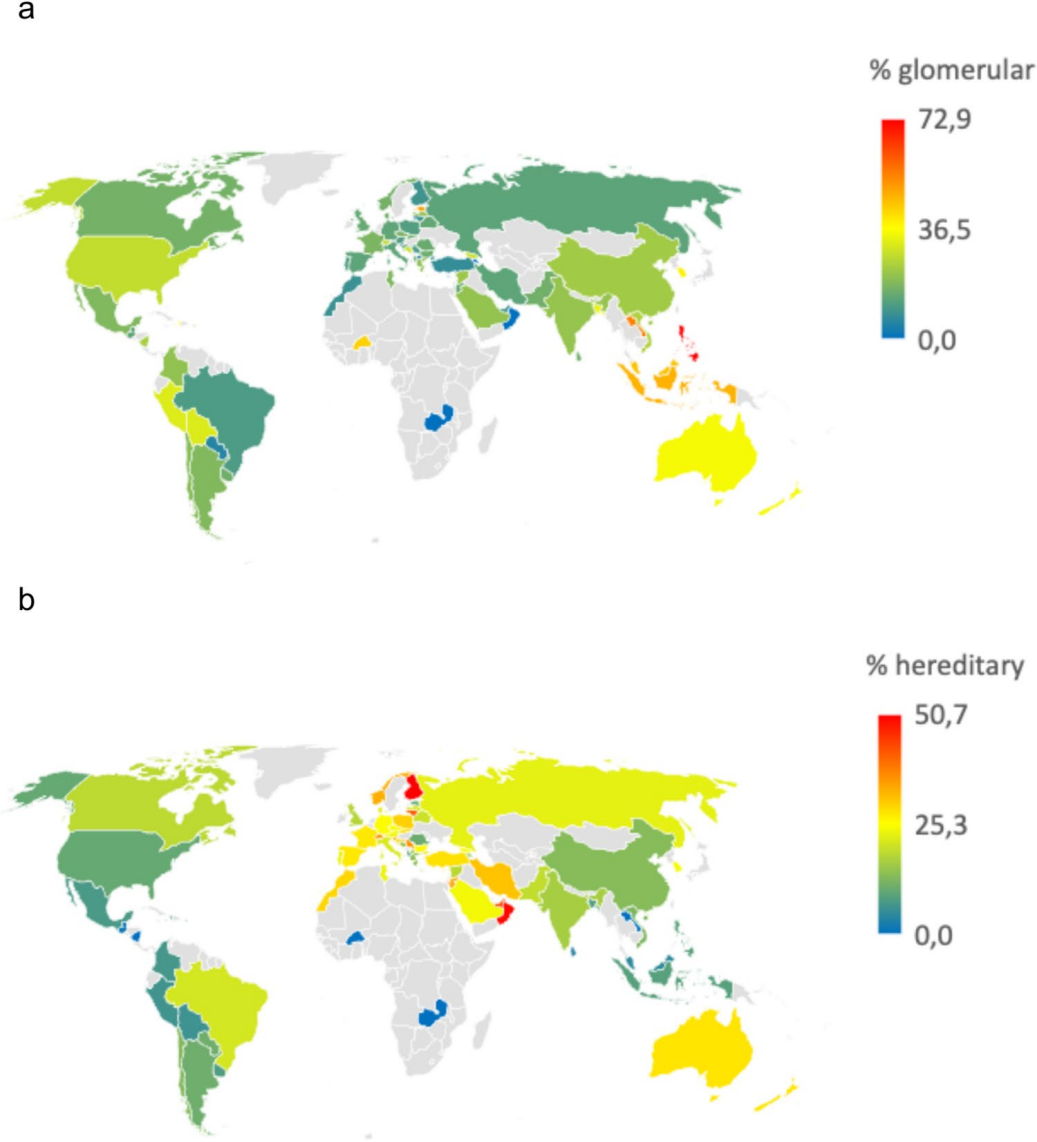
Relative proportion of glomerular (2**a**) and hereditary (2**b**) disorders causing KRT in childhood by country

Significant regional variation was noted in the relative frequency of acquired glomerulopathies and hereditary disorders. ***Immune-mediated glomerulopathies*** were most prevalent in Southeast Asia, accounting for 43% of pediatric KF cases, in contrast to 12–15% reported in Europe, Russia/Newly Independent States (NIS), the Middle East, and Africa (*p* < 0.001; Fig. 1, Table 2). The highest national proportions of glomerular diseases were observed in the Philippines, Laos, and South Korea (Fig. 2a).

**Fig. 3:**
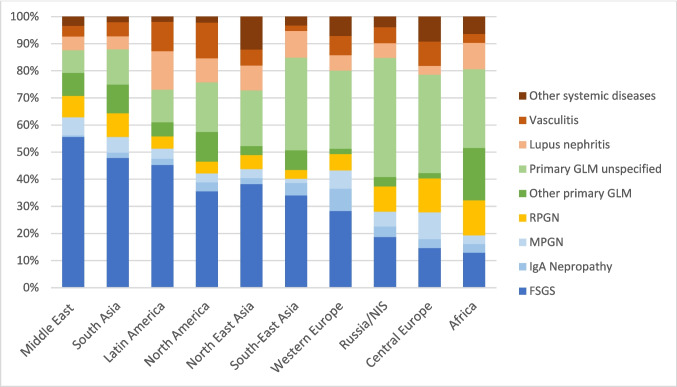
Distribution of glomerular diseases leading to kidney failure by global region

FSGS was the leading glomerular cause of KF, representing 34% of immune-mediated glomerulopathies globally, followed by vasculitis (8.3%) and systemic lupus erythematosus (SLE) nephritis (7.7%). The distribution of both primary and secondary glomerular disorders varied notably across regions. While FSGS was the predominant immune-mediated cause of KF in the Middle East (55%), South Asia (47%), North America (39%), and Northeast Asia (38%), its prevalence was considerably lower in Central Europe (14%). Lupus nephritis contributed most significantly to KF in Latin America (14%) and Southeast Asia (10%). IgA nephropathy accounted for 8% of KF cases in Western Europe, compared to only 2–4% in other regions (*p* < 0.001). A notably high proportion of cases (25%) were classified as unspecified primary glomerulopathies, with regional variation ranging from 8% in the Middle East to 44% in Russia/NIS (*p* < 0.001) (Fig. 3).

***Familial and hereditary kidney disorders*** (depicted by green shades in Fig. 1) were most common in the Middle East (30%), Africa (25%), and Europe (27%), and were least frequent in the Americas and Southeast Asia (10–12%). Finland, Saudi Arabia and Oman reported the highest fractions of hereditary disorders leading to KF (Fig. 2b). Across all regions cystic kidney diseases represented the leading hereditary cause of KF (44%), followed by hereditary and syndromic glomerulopathies. Hereditary tubulopathies were notably more prevalent in Africa (38%) and the Middle East (22%); they constituted the most common hereditary disorders in Tunisia (47%) and Pakistan (41%). Primary hyperoxaluria emerged as the predominant hereditary disorder leading to KF in these countries, accounting for 8 out of 9 cases in Tunisia and 72% of hereditary disorders in Pakistan.

***CAKUT*** disorders were the leading cause of KF in all regions except in North and Southeast Asia, where they accounted for only 19% and 25% of cases, respectively. Isolated kidney dysplasia was consistently reported as the most frequent form of CAKUT, comprising 35–60% of cases in most regions, and reaching up to 80% in Northeast Asia. In this latter region, obstructive uropathy was notably less common (6%) than in other areas (16–33%).

Considerable variation was also observed in the proportion of KF cases with ***unknown etiology***, which constituted 14% overall. This category was more prevalent in Northeast Asia, driven primarily by a high fraction of unexplained cases in China (41%). In Latin America, Guatemala stood out with 73% and Nicaragua with 56% unexplained cases.

## Factors associated with primary kidney disease (PKD) distribution

### Gross national income

**Fig. 4:**
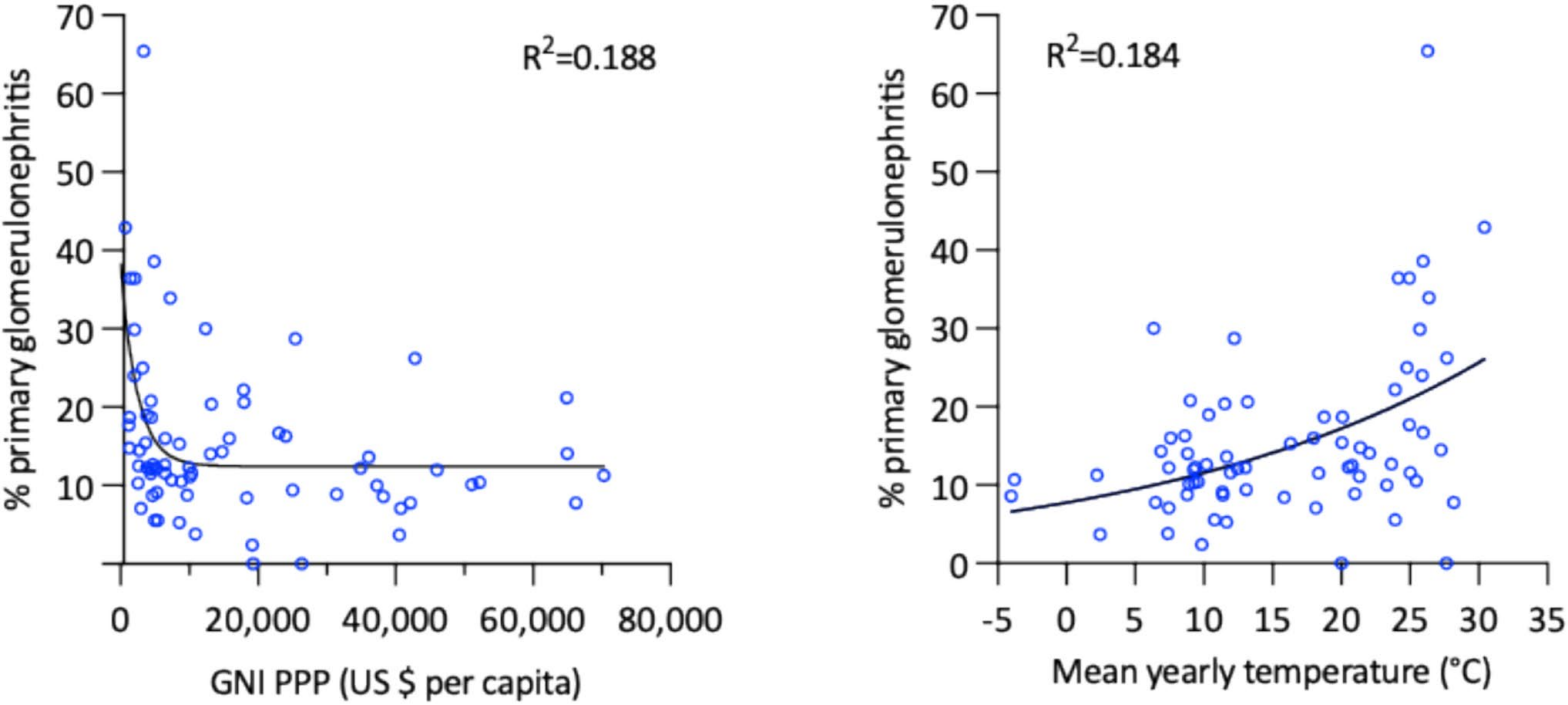
Factors associated with proportion of primary glomerular diseases as cause of kidney failure per country. *Left panel*: Gross national income (in US$, adjusted per purchasing power parity). *Right panel*: Average yearly temperature ((in °C)

Of the total cohort, 476 patients (2%) originated from low-income countries (LIC), 2,597 (11%) from low-middle-income countries (LMIC), 7,086 (30%) from high-middle-income countries (HMIC), and 13,461 (57%) from high-income countries (HIC). Significant differences were observed in the relative frequency of acquired immune-mediated glomerulopathies, which accounted for 43% of diagnoses in LIC as compared to 15% in countries with higher national incomes (*p* < 0.0001). Country-based comparison revealed a negative correlation between the fraction of immune-mediated glomerulopathies relative to all diagnoses and gross national income per capita (Fig. 4).

### Average yearly temperature

The reporting countries represented all climate zones with average yearly temperatures ranging from –4.0 °C in Canada to 30.4 °C in Burkina Faso. The average temperature was positively correlated with the fraction of immune-mediated glomerulopathies among all reported diagnoses per country (Fig. 4), whereas the frequency of IgA nephropathy was inversely correlated with temperature. None of the other diagnosis groups showed associations with ambient temperature.

When both GNI and temperature were included in a multiple linear regression model the effect of temperature on % primary glomerulopathies remained highly significant (p = 0.0004) whereas GNI lost significance (p = 0.288). However, significant negative interaction of the two parameters was found (GNI*temperature: P = 0.035), suggesting that at a given annual mean temperature, a lower GNI is associated with a higher prevalence of primary GN.

### Patient age

**Fig. 5:**
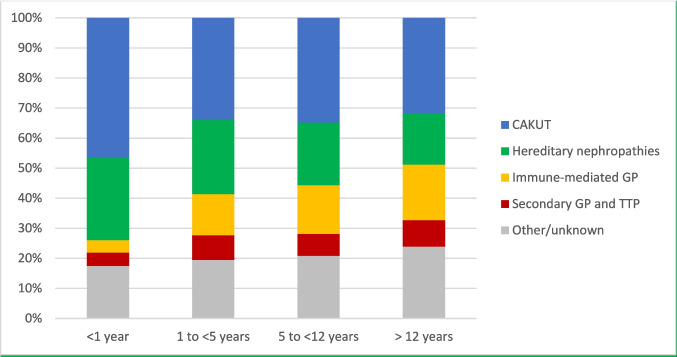
Primary kidney disease distribution by patient age

The distribution of KF etiologies by age group largely reflected the manifestation age and natural history of the diseases. CAKUT and hereditary nephropathies predominated among younger children, whereas immune-mediated glomerulopathies were more common in adolescents (Fig. 5). Post-ischemic CKD was observed to be twice as prevalent in children under five years of age compared to their older counterparts. “Unknown” and “other” causes of KF were most frequently reported in adolescent patients.

## Discussion

This study presents the first global, population-based comparative analysis of the disease spectrum leading to KF in childhood, leveraging registry data from multiple countries across all continents. Our investigation revealed substantial regional heterogeneity in disease distribution.

The most pronounced variation in disease occurrence was identified for **immune-mediated glomerulopathies**, which was a much less frequent cause of KF in Europe and Africa than in Asia and the Americas. The observed regional variation of glomerular disease etiologies is in keeping with findings of the International Kidney Biopsy Survey [17]. In an analysis of more than 42,000 mostly adult cases, FSGS predominated in North America, SLE nephritis in Asia and Latin America, and IgA nephropathy in Europe and Asia.

The high proportion of glomerular disorders particularly in Southeast Asia and parts of Latin America may be attributable to both genetic and environmental conditions. Genetic susceptibility to autoimmune conditions is known to vary by ethnicity. HLA variants enhancing autoantibody activity are more prevalent among Hispanic and Asian populations, which may exacerbate disease severity and accelerate progression to KF [18]. In addition, the high burden of bacterial, viral, and parasitic infections in regions characterized by hot and humid climatic conditions may trigger autoimmune disorders independently of genetic predisposition [19]. Indeed, we observed a significant association of the country-specific fraction of pediatric KF cases caused by primary glomerulonephritis with the average annual temperature.

Furthermore, limited healthcare resources may impact the risk of progression to KF of patients with treatable acquired glomerular conditions more than those with congenital and hereditary kidney disorders. In line with this notion, glomerular diseases were found to be 2.5-fold overrepresented as KF causes in low-income as compared to high- and upper-middle-income countries. Poor access to specialized care and effective immunosuppressive medications in low-resource settings may increase the likelihood of patients to progress to KF, and be represented in a KRT registry, even if the primary incidence of immune-mediated disorders was not greater than in higher-income regions. Notably in this context, our multivariate analysis suggested that environmental conditions are a stronger driving factor than economic wealth.

Notably, the highest prevalence of **FSGS** was reported in the Middle East. This finding is in keeping with a biopsy study in 376 Saudi Arabian children, where FSGS accounted for 32% of cases [20]. Given the high incidence of hereditary diseases in this region, it is likely that a major proportion of FSGS cases were not related to immune pathology but represented undiagnosed genetic podocytopathies. Likewise, the high FSGS prevalence reported for North America is likely due to African American patients who frequently harbor high-risk *APOL1* gene variants predisposing to FSGS and accelerated decline of kidney function [21].

The proportion of patients with KF due to **IgA nephropathy** was higher in Europe than East Asia, even though individuals of both East Asian and European descent are susceptible to IgA nephropathy. This discrepancy may reflect the widespread implementation of urinary screening programs in Eastern Asia, which has been demonstrated to cost-effectively reduce the incidence of KF among patients with IgA nephropathy [22, 23].

Hereditary, familial, and syndromic **kidney diseases of genetic origin** were most prevalent in the Middle East and North Africa, most likely reflecting cultural factors such as high rates of parental consanguinity common within Islamic societies. The relatively elevated frequency of genetic disorders reported by European centers may be attributed to broad access to advanced genetic diagnostics as well as the presence of large migrant populations, particularly from North Africa and the Middle East.

While **CAKUT** was the leading cause of pediatric KF globally, its relative prevalence exhibited marked regional variation, ranging from 18% in Northeast Asia to 46–47% in Latin America and Russia/NIS. Stratification by income level indicates that CAKUT is the predominant diagnosis in low-middle-income countries (LMICs), accounting for 55% of KF cases, compared to 45% in higher-income settings. Early diagnosis of CAKUT allows effective renoprotective management including prompt urological intervention, adequate fluid intake, prevention of urinary tract infections, and pharmacological RAS blockade. Data from high-income European countries indicate that 50% of children diagnosed with CAKUT do not require KRT within the first 30 years of life [24]. In contrast, access to early diagnosis and therapeutic interventions may be more limited in LMICs, resulting in faster progression and a higher fraction of children on KRT in these countries. The relatively low proportion of CAKUT cases reported in the lowest income group may reflect elevated mortality rates associated with missed or late diagnosis. Additionally, limited healthcare infrastructure, insufficient government policies, and shortages of pediatric nephrologists substantially restrict access to KRT in low-income settings particularly for infants, resulting in markedly reduced treatment rates [25]. In a recent study, the proportion of infants initiating KRT ranged from 1–2% in Asia and Africa to 12% in Western Europe [26].

Notably, a considerable proportion of KF cases across all regions and national income levels were of **unknown etiology**. High rates of KF patients with unidentified etiology likely reflect the often asymptomatic progression of CKD to advanced stages, compounded by global inequities in nephrology care and delayed referrals to specialized care [27]. The observation also may indicate significant gaps in current diagnostic capabilities and variability in the extent of diagnostic workup across countries. Several measures could be cost-efficient in improving the timely detection of CKD in children. The existence of fetal and neonatal ultrasound programs is key to the early detection of CAKUT cases [28]. Routine implementation of genetic testing has been shown to clarify the underlying disease in up to 40% of pediatric KF cases and enhance clinical management [29]. Urine dipstick screening is an inexpensive and efficient tool for early detection of glomerular disorders which, given the availability of effective therapies, may justify the introduction of national screening programs in countries with a high incidence of glomerular diseases leading to KF [30]. In some circumstances, regional clustering of kidney failure from unknown etiology might be related to unidentified environmental factors. This is exemplified by the high proportion of patients with unknown etiology reported for Guatemala and Nicaragua, countries that represent a hotspot of Mesoamerican Endemic Nephropathy, a condition assumed to be related to an unidentified environmental toxin or infectious agent [27, 31].

Several limitations inherent to this study warrant consideration. Data collection methodologies varied, with some regions contributing data from national registries and others from individual centers. Moreover, the data from different registries have been collected over various time periods. Participation in the IPDN registry and the web-based part of the IPNA registry is voluntary, introducing potential selection bias. In addition, adolescents aged 16–17 years may be underrepresented in some countries, as they are often treated in adult care settings. Globally, regions lacking resources for registry documentation are underrepresented in this analysis. Also, the classification of regions according to the ISN regional boards may have oversimplified the ethnic composition and macroeconomic conditions within specific geographic areas. Furthermore, regional differences in access to and indication policies for kidney biopsies and genetic testing might have biased the reported disease etiologies. Finally, access to KRT may vary by age and underlying disease, particularly in low-resource environments where chronic KRT may be available to adolescents but not to younger children due to the lack of pediatric nephrology services [25]. Consequently, the underlying diseases of neonates, young infants and patients with severe syndromic disorders who were not accepted for KRT may have been regionally underrepresented as causes of KF. Variable representation of the youngest, CAKUT-predominant age group would impact reciprocally on the calculated regional proportions of underlying kidney diseases.

Despite these limitations, this study provides valuable insights that may inform future CKD screening programs, guide national healthcare resource allocation and disease management and help prioritize target regions for clinical trials. The study’s strengths include its large, diverse dataset encompassing multiple countries and regions. Future enhancements to the registry could incorporate detailed biopsy data, biomarker analyses, genetic testing results, and comprehensive individual-level socioeconomic and healthcare access information.

In conclusion, significant global variation exists in the etiologies of pediatric KF, influenced by geographic, ethnic, genetic, and socioeconomic factors. A substantial proportion of KF cases remain unexplained, even in high-income countries, underscoring persistent gaps in early detection of pediatric kidney disease worldwide. The IPNA pediatric KRT registry establishes a foundation for life-course epidemiological studies aimed at identifying at-risk populations and enhancing early diagnosis and management strategies.

## Supplementary Information

Below is the link to the electronic supplementary material.

Graphical abstract (PPTX 246 KB)

Supplementary file2 (DOCX 30 KB)

## Data Availability

The data underlying this manuscript cannot be shared with any third party because the national and international registries that provided data to the IPNA Registry remain the owners of the data.

## Declarations

### Collaborators

*IPNA Registry Participants:* Hazem Awad, Loai Akram Eid, Eihab Al Khasawneh, Gurinder Kumar, Watfa Shahwan Al Dhaheri, Helen Nazaryan, Maria Laura Beaudoin, Saimul Haque, Azmeri Sultana, Gerard Coulibaly, Claudia Gorena, Reyna Callisaya, Clotilde Garcia, Oreste Ferra Neto, Francisco Cano, Hong Xu, Shenqian Jing, Yihui Zhai, Eugene Chan, Richard Baquero Rodriguez, Hesham Safouh, Tinatin Davitaia, Maria Lipka, Cristina Zelaya, Judith Exantus, Henny Adriani Puspitasari, Choni Rinat, Vardit Peles, Aditi Sinha, Alpana Ohri, Kumar Manish, Manoj Matnani, Saumil Gaur, Sushmita Banerji, Jyoti Sharma, Suprita Kalra, Susan Uthup, Nivedita Kamath, Uma Ali, Amol Mbdave, Swati Bhardwaj, Neha Agarwal, Mehul Shah, Puneet Singh, Aliza Mittal, Sukanya Govindan, Pankaj Deshpande, Indira Agarwal, Vaibhav Keskar, Sudha Ekambaram, Sukanya Govindan, Satya Prasad VVR, Koushal Kumar Khajuria, Kinnari Vala, Kalaivani Ganesan, Kalaivani Ganesan, Subal Pradhan, Nakysa Hooman, Afshin Safaie Asl, Hamidreza Badeli, Hadi Sorkhi, Hamid Mohamadjafari, Ahmadali Nikibakhsh, Ali Derakhshan, Mitra Basiratnia, Masoumeh Mohkam, Mostafa Sharifian Dorcheh, Nasrin Esfandiar, Seyyed Mohammad Taghi Hosseini Tabatabaei, Daryoush Fahimi, Fahimeh Askarian, Mastaneh Moghtaderi, Neamatollah Ataei, Seyed Taher Esfahani, Nahid Mamizadeh, Parsa Yousefichaijan, Rama Naghshizadian, Anoush Azarfar, Fatemeh Ghane Sharbaf, Saeideh Parvaresh, Ehsan Valavi, Mojgan Mazaheri, Mohammad Reza Razavi, Mohsen Akhavan Sepahi, Hossein Emad Momtaz, Abolhassan Seyedzadeh, Simin Sadeghi Bojd, Maryam Esteghamati, Banafsheh Arad, Mahmood Maleknejad, Masoudeh Sabzechian, Alaleh Gheissari, Zahra Noparast, Reham Al Mardini, Ghazi Mohamad Al-Salaita, Hee Gyung Kang, Philavanh Kedsadtha, Aoun Bilal, Sami Sanjad, Umeshi Karu, Kenza Soulami, Yap Yok Chin, Adebowale Ademola, Mohamed Alriyami, Reyner Loza, Marbella Angeles, Alona R. Arias-Briones, Madiha Aziz, Seema Hashmi, Khem Chand Moorani, Naureen Akhtar, Muhammad Imran, Farhana Amanullah, Iftikhar Ijaz, Ahad Qayyum, Marta Suarez, Marlene Martinez Pico, Mohammed Shalaby, Nada Kalakattawi, Najlaa Alotaibi, Osama Safder, Sherif Mohamed ElDesoky, Layla Abdullah Basheer, Jameela Abdulaziz Kari, Mya Than, Ng Kar Hui, Bassam Saeed, Hala Wannous, Huda Mazloum, Ghalia Sawaf, Sameh Mabrouk, Anthony Batte, Anthony Batte, Anabella Rebori, Claudia Vieira, Vu Huy Tru, Chisambo Mwaba.

*Collaborative ESPN/ERA Registry and IPNA Registry Participants:* D Shtiza, G Mayer, J Kerschbaum, L Buchwinkler, D Kaiser-Feistmantl, S Baiko, O Raikevic-Liachovskaya, A Dudarevich, D Pokrajac, D Roussinov, I Kos, I Jakopcic, J Ivancic, M Ban, Matkovic, M Davidovic, L Lamot, K Vrljičak, A Elia, K Vondrak, K Hommel, Ü Toots, J Helve, P Finne, C Couchoud, M Lassalle, J Hogan, K Krupka, B Höcker, L Pape, B Tönshoff, T Davitaia, G Moustakas, G Reusz, O Horváth, Cs Berecki, A Szabó, T Szabó, O Lakatos, B Gianoglio, I Guzzo, E la Porta, F Paglialonga, C Corrado, E Vidal, E Verrina, A Popova, S Derkevica, V Kuzema, A Jankauskiene, S Rudaitis, V Said-Conti, N Abazi, A Åsberg, AV Reisæter, A Bjerre, A Zurowska, I Zagozdzon, C Mota, JE Esteves, M Abranches, C Gomes, G Mircescu, L Garneata, E Podgoreanu, EA Molchanova, EV Zakharova, AM Andrusev, M Kostić, B Spasojević, M Cvetković, I Gojković, D Paripović, G Miloševski-Lomić, L Podracka, G Kolvek, N Battelino, G Novljan, J Buturovic-Ponikvar, A Alonso Melgar and the Spanish Paediatric Registry, E Maurer, GF Laube, CE Kuehni, P Parvex, S Tschumi, L Mader, S Bakkaloglu, SP Fomina, All the staff of the UK Renal Registry and of the renal units submitting data, and All of the Scottish Renal Registry team.

*IPDN Registry Participants:* Laura Alconcher, Patricia G Vallés, Monica Grossman, Paula Alejandra Coccia, Rosana Salim, Sara Testa, Enrico Verrina, Karel Vondrack, Lorena Sànchez Barbosa, Nikoleta Printza, Constantinos J. Stefanidis, Attila Szabo, Rukshana Shroff, Amrit Kaur, Judith Hayes, Chris Reid, Claus Peter Schmitt, Susanne Schaefer, Julia Thumfart, Lars Pape, Marcus Kemper, Günter Klaus, Rainer Buescher, Sandra Habbig.

The ESPN/ERA Registry, providing data from European countries to the IPNA KRT registry, is funded by the European Society of Pediatric Nephrology (ESPN) and the European Renal Association (ERA).

Support was also received from ERKNet, the European Reference Network for Rare Kidney Diseases. ERKNet is funded by the European Union within the framework of the EU4Health program (101085068).
