# Supplementary material for: Regional variation of underlying kidney diseases in children undergoing chronic kidney replacement therapy around the globe
Source: Pediatr Nephrol. 2025 Dec 8;41(5):1451–63. doi: 10.1007/s00467-025-07096-3 (PMC13009119; doi:10.1007/s00467-025-07096-3)
Supplement: Supplementary file 1 — Graphical abstract (PPTX 246 KB) [file 467_2025_7096_MOESM1_ESM.pptx]

## Slide 1
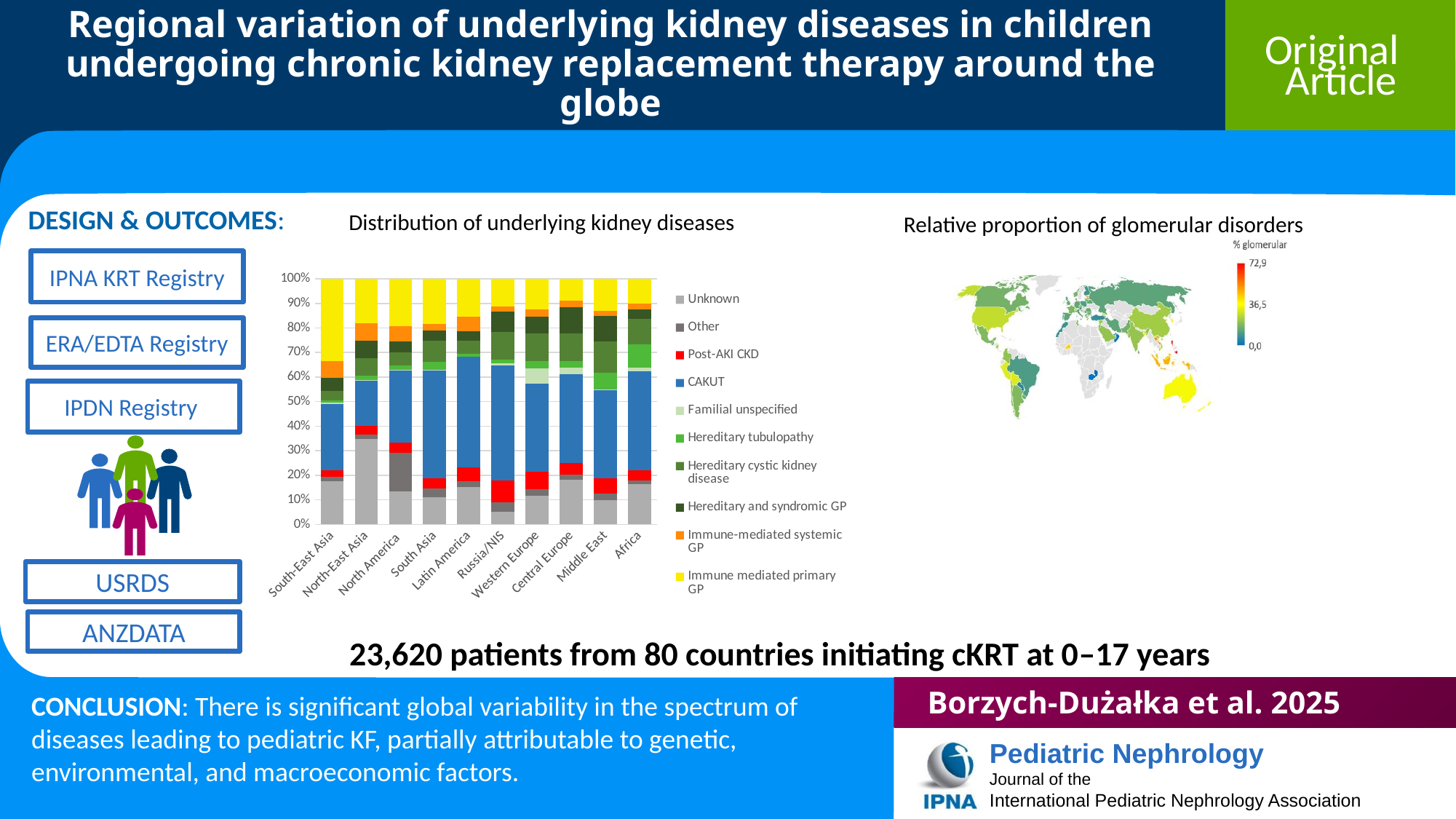

Regional variation of underlying kidney diseases in children undergoing chronic kidney replacement therapy around the globe
DESIGN & OUTCOMES:
Distribution of underlying kidney diseases
Relative proportion of glomerular disorders
IPNA KRT Registry
### Chart
| Category | Unknown | Other | Post-AKI CKD | CAKUT | Familial unspecified | Hereditary tubulopathy | Hereditary cystic kidney disease | Hereditary and syndromic GP | Immune-mediated systemic GP | Immune mediated primary GP |
|---|---|---|---|---|---|---|---|---|---|---|
| South-East Asia | 260.0 | 28.0 | 38.0 | 401.0 | 8.0 | 12.0 | 58.0 | 81.0 | 101.0 | 496.0 |
| North-East Asia | 377.0 | 20.0 | 38.0 | 202.0 | 2.0 | 18.0 | 79.0 | 76.0 | 77.0 | 197.0 |
| North America | 699.0 | 829.0 | 219.0 | 1527.0 | 23.0 | 80.0 | 288.0 | 227.0 | 331.0 | 1004.0 |
| South Asia | 191.0 | 57.0 | 75.0 | 749.0 | 3.0 | 55.0 | 149.0 | 69.0 | 48.0 | 313.0 |
| Latin America | 182.0 | 29.0 | 68.0 | 541.0 | 0.0 | 14.0 | 63.0 | 47.0 | 73.0 | 184.0 |
| Russia/NIS | 101.0 | 70.0 | 176.0 | 899.0 | 18.0 | 32.0 | 215.0 | 161.0 | 39.0 | 218.0 |
| Western Europe | 705.0 | 156.0 | 425.0 | 2167.0 | 382.0 | 169.0 | 691.0 | 411.0 | 184.0 | 743.0 |
| Central Europe | 625.0 | 79.0 | 159.0 | 1249.0 | 92.0 | 95.0 | 392.0 | 362.0 | 90.0 | 312.0 |
| Middle East | 115.0 | 30.0 | 76.0 | 418.0 | 5.0 | 79.0 | 147.0 | 122.0 | 25.0 | 153.0 |
| Africa | 39.0 | 4.0 | 10.0 | 97.0 | 3.0 | 23.0 | 25.0 | 9.0 | 6.0 | 24.0 |ERA/EDTA Registry
IPDN Registry
USRDS
ANZDATA
23,620 patients from 80 countries initiating cKRT at 0–17 years
Borzych-Dużałka et al. 2025
CONCLUSION: There is significant global variability in the spectrum of diseases leading to pediatric KF, partially attributable to genetic, environmental, and macroeconomic factors.
