# Supplementary material for: Regional variation of underlying kidney diseases in children undergoing chronic kidney replacement therapy around the globe
Source: Pediatr Nephrol. 2025 Dec 8;41(5):1451–63. doi: 10.1007/s00467-025-07096-3 (PMC13009119; doi:10.1007/s00467-025-07096-3)
Supplement: Supplementary file 2 — Supplementary file2 (DOCX 30 KB) [file 467_2025_7096_MOESM2_ESM.docx]

**Supplemental Table1.** Mapping table of primary kidney diseases by Registry.

| **Category** | **Subcategory** | **IPNA Registry** | **IPDN Registry** | **USRDS Registry** | **ANZDATA Registry** |
| --- | --- | --- | --- | --- | --- |
| **Congenital Abnormalities of Kidney and Urinary Tract** | Kidney A/hypo/dysplasia | Dysplastic kidney  Multicyclic dysplastic kidney  Prune Belly Syndrome  Oligomeganephronia | A/hypo/dysplasia  Prune Belly Syndrome | Renal hypoplasia, dysplasia, oligomeganephronia | Hypodysplasia/Dysplasia |
|  | Obstructive uropathy | Obstructive uropathy (incl. PUV) | Obstructive uropathy | Congenital obstructive uropathies | Posterior Urethral Valves |
|  | Reflux nephropathy | Reflux nephropathy  Megacystis megaureter | Reflux nephropathy | Reflux nephropathy | Reflux Nephropathy |
|  | Other CAKUT | Neurogenic bladder  Bladder exstrophy  Other structural abnormalities  Branchio-oto-renal-syndrome  Medullary sponge kidney  Renal coloboma  Glomerulocystic disease |  |  |  |
| **Immune-mediated glomerulonephritis** | Primary glomerulonephritis | Primary GLM not further specified  FSGS  IgA Nephropathy  Idiopathic RPGN  Minimal change disease  MPGN t.1  MPGN t.2  Membranous nephropathy  MPGN not further specified  Diffuse endocapillary (post-infectious) GLM  IgA nephropathy  IgM nephropathy  MPGN t.3 | FSGS  MPGN t.1  MPGN t.2  Membranous nephropathy  Idiopathic RPGN  Chronic Glomerulonephritis  IgA nephropathy | FSGS  MPGN t.1  MPGN t.2  Membranous nephropathy  Glomerulonephritis histologically not examined  IgA nephropathy  GLM with lesion of RPGN | Glomerular disease  FSGS |
|  | Secondary GLM | Lupus erythematosus  Hypertensive nephropathy  Non further specified vasculitis  Henoch-Schoenlein Purpura  Granulomatosis with polyangiitis  Goodpasture Syndrome  ANCA (+) vasculitis  Amyloidosis  Diabetic nephropathy  Polyarthritis nodosa  Microscopic polyangiitis  ANCA (-) vasculitis  Cryoglobulinemia (essential or secondary)  Non further specified systemic disease  Scleroderma  Eosinophilic granulomatosis with polyangiitis | Systemic Inflammatory Disease (SID) – Lupus nephritis  SID-Henoch-Schoenlein purpura  SID – Wegener granulomatosis  SID-other  Diabetic glomerulopathy | Lupus erythematosus  Henoch-Schoenlein Purpura  Polyarthritis  Wegener Granulomatosis  Goodpasture syndrome  Scleroderma  Diabetes with renal manifestations, Type 2  Diabetes with renal manifestations, Type 1 | Diabetic Kidney Disease |
| **Hereditary/familial kidney diseases** | Cystic | ADTKD  ARPKD  Cystic kidney disease – unspecified  Nephronophthisis  ADPKD  Bardet-Biedl Syndrome  PKD unspecified  Tuberous sclerosis | Polycystic Kidney Disease  Medullary cystic disease (nephronophthisis) | Polycystic kidneys, adult type (dominant)  Polycystic kidneys, infantile (recessive)  Medullary cystic disease, including nephronophthisis  Tuberous sclerosis | Polycystic Kidney Disease  Nephronophthisis/ADTKD |
|  | Glomerulopathies | Congenital nephrotic syndrome  Alport Syndrome  Familial FSGS (AR)  Denys-Drash Syndrome  Fraser Syndrome  Thin basement membrane disease | Congenital nephrotic syndrome  Familial nephritis (Alport Syndrome)  Drash Syndrome | Hereditary nephritis, Alport syndrome | Congenital nephrotic syndrome  Alport Syndrome |
|  | Hereditary tubulopathies | Cystinosis  Primary hyperoxaluria  Renal Tubular Acidosis  Barter Syndrome  Fanconi Syndrome  Lowe Syndrome  Familial hypomagnesemia  Dent Disease  Hypophosphatemic rickets  Fabry Disease | Cystinosis  Oxalosis | Cystinosis and disorders of amino acid transfer  Primary oxalosis | Cystinosis |
|  | Other familial kidney diseases | Familial unspecified |  |  |  |
| **Post AKI CKD (including thrombotic microangiopathies)** |  | Atypical HUS  Typical HUS  HUS not specified  Secondary HUS  Familial/congenital HUS  TTP  Ischemic acute kidney injury (ACN)  Atheroembolic renal disease  Renal artery stenosis | HUS  Renal venous thrombosis | Haemolytic uremic syndrome  Thrombotic microangiopathy  Renal artery stenosis  Cholesterol emboli, renal emboli  Large vessel disease  Tubular necrosis  (no recovery) | Haemolytic Uraemic Syndrome  Cortical Necrosis |
| **Other (including:**  **Tubulointerstitial nephritis**  **Toxic/infectious**  **Oncologic**  **Post-traumatic)** |  | TIN not further specified  Urolithiasis  Pyelonephritis  TIN secondary to metabolic disease  TIN autoimmune  TINU  Aristolochic acid nephropathy  Urate nephropathy  Medications induced nephropathy  Unspecified infections  Tuberculosis  Leptospirosis  Nephrotoxicity (not specified)  HIV  Sickle cell Disease  Unspecified kidney malignancies  Wilms’s tumour  Other malignancy | Willms’ tumor  Renal infarct  Sickle cell nephropathy | Analgesic abuse  Nephropathy due to drugs and biological substances  Nephrolithiasis  Acquired obstructive uropathy  Chronic interstitial nephritis  Acute interstitial nephritis  Other disorders of calcium metabolism  Sickle cell disease/anemia  Sickle cell trait and other sickle cell (HbS/Hb other)  AIDS nephropathy  Renal tumor (malignant)  Renal tumor (unspecified)  Lymphoma of kidneys  Amyloidosis  Complications of transplanted organ  Traumatic or surgical loss of kidney(s)  Hepatorenal syndrome  Unspecified with renal failure | Interstitial Nephritis  Misc/Other |
| **Unknown** |  | Unknown | Unknown | Uncertain | Uncertain  Not reported |

**Supplemental Table 2.** Enrolment year by region and country.

|  | Africa | Latin America | Western Europe | Eastern and Central Europe | Russia and Newly Independent States | Middle East | Northeast Asia | South Asia | South-East Asia & Oceania | North America  And Caribbean |
| --- | --- | --- | --- | --- | --- | --- | --- | --- | --- | --- |
| **Year of enrollment** | 2001-2025 | 1998-2024 | 1997-2024 | 1997-2023 | 1997-2022 | 1999-2023 | 1998-2024 | 1997-2024 | 1997-2024 | 1997-2022 |
| **Year of enrolment by country** | Burkina Faso  (2010-2017)  Egipt  (2020)  Marocco  (2001-2024)  Nigeria  (2024-2025)  Tunisia  (2007-2021)  Uganda  (2018)  Zambia  (2017-2018) | Argentina (2000-2024)  Bolivia  (2011-2017)  Brazil  (2005-2022)  Chile  (2000-2018)  Colombia  (2008-2024)  Guatemala  (2020-2023)  Haiti  (2015-2024)  Mexico  (1998-2010)  Nicaragua  (2005-2013)  Peru  (2001-2020)  Paraguay  (2009-2018)  Uruguay  (2010-2022) | Austria (1997-2019)  Denmark  (1997-2019)  Finland  (1997-2019)  France  (1997-2019)  Germany (1997-2024)  Greece  (1998-2020)  Israel  (1997-2024)  Italy  (1997-2023)  Malta  (2003-2017)  Norway  (1997-2019)  Portugal  (1997-2019)  Spain  (1997-2019)  Switzerland  (1997-2018)  UK  (1998-2024) | Albania  (2008-2019)  Bosnia &  Herzegovina  (1998-2018)  Croatia  (1997-2017)  Bulgaria  (1998-2019)  Cyprus  (2002-2019)  Czech Rep  (2002-2023)  Estonia  (1999-2017)  Hungary  (2003-2017)  Latvia  (2009-2019)  Lithuania  (1999-2019)  Macedonia  (2001-2019)  Poland  (1997-2019)  Romania  (1997-2019)  Serbia  (1997-2019)  Slovenia  (1997-2018)  Slovakia  (1997-2019)  Turkey  (1998-2019) | Armenia  (2011-2022)  Belarus  (2003-2019)  Georgia  (2016-2020)  Russia  (1997-2019)  Ukraine  (2002-2019) | Jordan  (2006-2018)  Syria  (2004-2021)  Iran  (1999-2023)  Lebanon  (2017-2018)  Oman  (2012-2021)  Saudi Arabia (2012-2021)  United Arab Emirates (2009-2023) | China  (2004-2022)  Hong Kong (1998-2023)  South Korea (2001-2024) | Bangladesh (2005-2024)  India  (1997-2024)  Sri Lanka  (2004-2018)  Pakistan  (2007-2023) | Indonesia  (2015-2024)  Laos  (2012-2023)  Malaysia  (1997-2020)  Philippines  (2010-2024)  Singapore  (1999-2021)  Vietnam  (2018-2019)  Australia/New Zealand  (2017-2022)** | USA  (2018-2022)*  Canada  (1997-2022)  Puerto Rico  (2005-2018) |
